# On Life Saving from Drowning by Self Inflation

**Published:** 1885-05

**Authors:** 


### On Life Saving From Drowning by Self-Inflation.

Dr. Henry Silvester, who is widely known as the author of the best method of resuscitation of the apparently drowned, publishes in the *Lancet* of January 3, 1885, a method which he claims will prevent persons from drowning. The method which he proposes is to distend the skin of the neck and upper part of the chest sufficiently with air to support the weight of the body when immersed, the inflation being effected by the person himself by means of his lungs without the intervention of appliances. The necessary operation consists in making a small puncture, not larger than necessary to allow of the passage of a small blow-pipe, in the mucous membrane of the mouth, the object being to open a communication for the passage of air from the cavity of the mouth into the subcutaneous space of the neck. The situation chosen for the puncture is in the angle formed between the gum of the lower jaw and the side of the under lip or cheek, about opposite the first molar tooth of the lower jaw. The point of the instrument perforating should be passed down a short distance between the skin of the side of the face and the superficial facia of the neck, its point being guided by the finger placed on the outside of the face and neck, taking care not to puncture either the skin or the superficial fascia. This having been done and the instrument removed, in order to inflate the skin of the neck and the chest, the person should close the mouth and the nose, and make a succession of forcible expiratory efforts, when the air contained in the cavity of the mouth will pass freely into the subcutaneous tissue of the neck. These expiratory efforts, inspiration being effected through the nostrils, should be continued until the skin is fully distended with air, which will pass readily to both sides of the neck and down the chest as far as the nipples ; and this is all that is required to render the body bouyant in water. Should it so happen that the superficial facia has been punctured and the air pass beneath it, the only difference in effect would be that the extent of air would be limited by the attachments of that membrane to the clavical below and the border of the jaw above. The amount of air which the skin of the average neck is capable of holding without undue detension has been measured, and found to be enough to support ten pounds, and is amply sufficient to support the body immersed in water. The time required for inflation is found to be less than three minutes. The neck may be kept in an inflated condition by closing the puncture by pressure on the outside of the cheek by the finger, or by keeping the mouth distended with air ; and when required the air may be immediately discharged from the neck by allowing the puncture to remain open, or by suction.

The advantages which Dr. Silvester claims for this method may be summed up as follows :

1. The proceeding is perfectly harmless and almost painless, quickly done and almost immediately recovered from.

2. It may be learned in a few minutes, no technical knowledge being required, and may be accomplished by the person himself without assistance.

3. No special apparatus is required. In a emergency the point of a pen-knife, or even a sharp-pointed splinter of wood, is all that would be required. The inflating apparatus is the person's own lungs.

4. The air could be repeatedly re-inflated, and even during prolonged immersion.

